# Reducing routine vaccination dropout rates: evaluating two interventions in three Kenyan districts, 2014

**DOI:** 10.1186/s12889-016-2823-5

**Published:** 2016-02-16

**Authors:** Adam Haji, S. Lowther, Z. Ngan’ga, Z. Gura, C. Tabu, H. Sandhu, Wences Arvelo

**Affiliations:** College of Health Sciences, Jomo Kenyatta University of Agriculture and Technology, Nairobi, Kenya; Field Epidemiology and Laboratory Training Program, Kenya Ministry of Health, Nairobi, Kenya; Global Immunization Division, US Centers of Disease Control, and Prevention, Nairobi, Kenya; Division for Global Health Protection, US Centers of Disease Control and Prevention, Nairobi, Kenya; Division of Vaccines and Immunization, Kenya Ministry of Health, Nairobi, Kenya

**Keywords:** SMS, Sticker, Dropout, Reminder, Vaccination

## Abstract

**Background:**

Globally, vaccine preventable diseases are responsible for nearly 20 % of deaths annually among children <5 years old. Worldwide, many children dropout from the vaccination program, are vaccinated late, or incompletely vaccinated. We evaluated the impact of text messaging and sticker reminders to reduce dropouts from the vaccination program.

**Methods:**

The evaluation was conducted in three selected districts in Kenya: Machakos, Langata and Njoro. Three health facilities were selected in each district, and randomly allocated to send text messages or provide stickers reminding parents to bring their children for second and third dose of pentavalent vaccine, or to the control group (routine reminder) with next appointment date indicated on the well-child booklet. Children aged <12 months presenting for their first dose of pentavalent vaccine were enrolled. A dropout was defined as not returning for vaccination ≥2 weeks after scheduled date for third dose of pentavalent vaccine. We calculated dropout rate as a percentage of the difference between first and third pentavalent dose.

**Results:**

We enrolled 1,116 children; 372 in each intervention and 372 controls between February and October 2014. Median age was 45 days old (range: 31–99 days), and 574 (51 %) were male. There were 136 (12 %) dropouts. Thirteen (4 %) children dropped out among those who received text messages, 60 (16 %) among who received sticker reminders, and 63 (17 %) among the controls. Having a caregiver with below secondary education [Odds Ratio (OR) 1.8, 95 % Confidence Interval (CI) 1.1–3.2], and residing >5 km from health facility (OR 1.6, CI 1.0–2.7) were associated with higher odds of dropping out. Those who received text messages were less likely to drop out compared to controls (OR 0.2, CI 0.04–0.8). There was no statistical difference between those who received stickers and controls (OR 0.9, CI 0.5–1.6).

**Conclusion:**

Text message reminders can reduce vaccination dropout rates in Kenya. We recommend the extended implementation of text message reminders in routine vaccination services.

## Background

Globally, vaccine preventable diseases are responsible for nearly 20 % of the 8.8 million deaths annually among children under five years of age [1]. Despite documented benefits that vaccines are efficient and cost-effective interventions for improving child survival, children in many parts of the world, particularly in Sub-Saharan Africa, are either vaccinated late or unvaccinated all together [2, 3]. In Kenya the first dose pentavalent vaccine, a combined routine childhood vaccine against diptheria, pertussis, tetanus, hepatis B and Haemophilus influenzae type b is given at six weeks of age, a second dose at ten weeks and a third dose at fourteen weeks or at first contact with four weeks apart between doses. The coverage for the third dose of pentavalent vaccine has increased at the national level from 63 % in 2000 to 84 % in 2013 [4]. Despite improvements of national coverage, many districts in Kenya continue to report low vaccination coverage. In 2013, only 45 % of the districts attained ≥80 % coverage for the third dose of pentavalent vaccine [4]. Additionally, vaccination dropout rates still remain high with over 27 % of Kenyan districts reporting dropout rates between 10 %–33 % for the third dose of pentavalent vaccine in 2011 [5]. Low vaccination coverage is associated with outbreaks of vaccine preventable diseases.

Several interventions have been used to reduce dropout rates for vaccinations among children. Sticker reminders with recommended return dates for vaccination placed strategically within the home have been shown to reduce vaccination dropouts in Ethiopia [6]. Postcards, automated telephone or mail reminders and outreach services have also been documented to improve vaccination coverage [7]. Mobile phones are increasingly being used for health applications [8–12], such as improving vaccination coverage [13, 14], promoting adherence to drug treatments for chronic diseases [15, 16], increasing uptake of screening tests [17–20], improving clinical appointment attendance [21, 22] and providing training health workers in malaria treatment [23]. Short message services (SMSs) through mobile phones have been successfully used to reduce dropout for vaccination services in Zimbabwe [24].

There has been limited research in Kenya comparing use of SMS or sticker reminders to improve vaccination coverage and reduce vaccination dropouts. One study conducted in Western Kenya with a small sample size showed significant benefits of SMS for reducing dropouts, but results may have been biased by monetary compensation of participants [25]. Evidence is needed to corroborate the effectiveness of of SMS or sticker reminders in routine vaccination programs throughout the country. We evaluated the impact of SMS and sticker reminders to reduce dropout rates for routine childhood vaccinations, and determined factors associated with missed vaccination in selected districts in Kenya.

## Methods

### Study sites

**Fig. 1:**
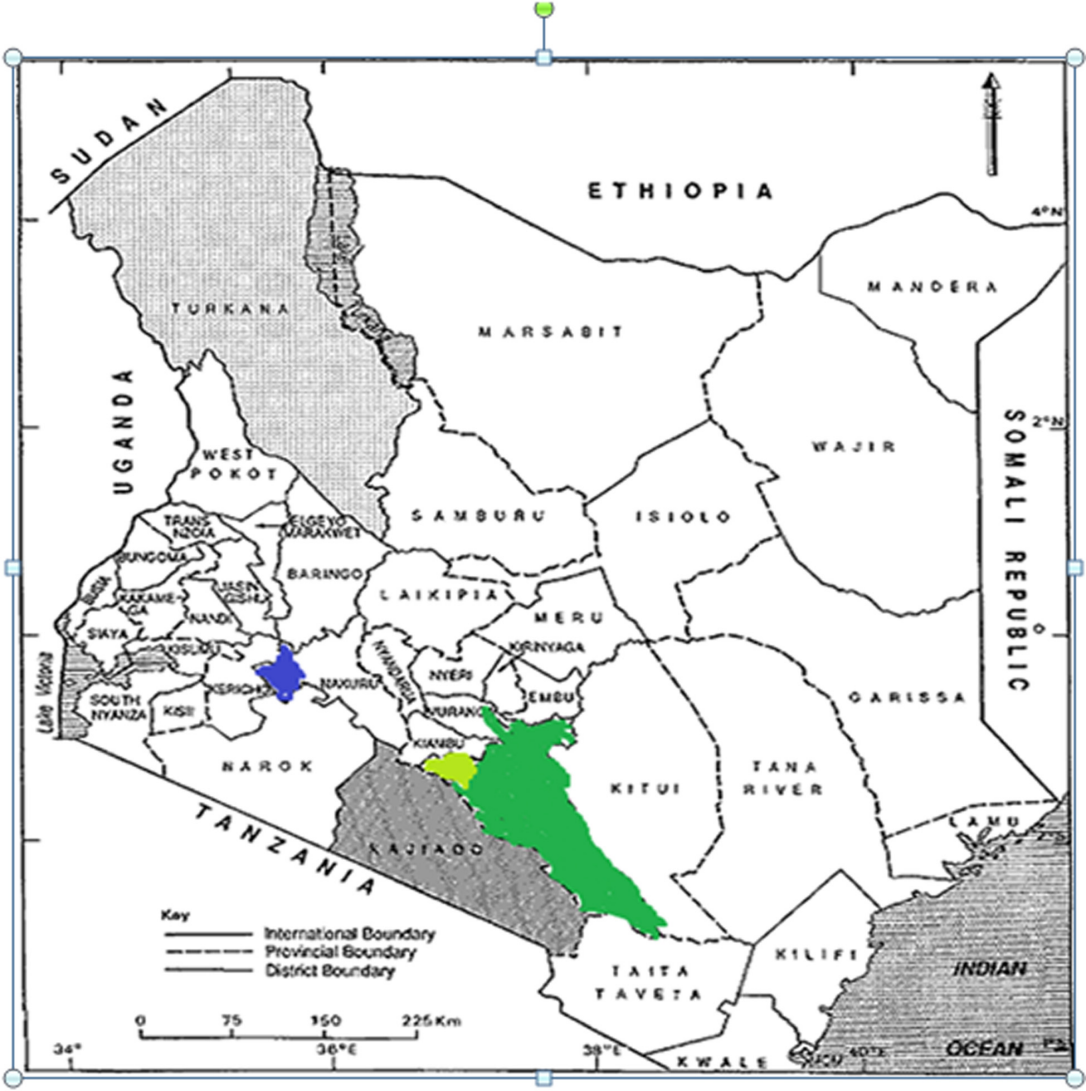
Map of Kenya showing study sites

We conducted an evaluation study in three selected districts in Kenya. District selection was based on pentavalent vaccine coverage for 2012. Districts with more than 10 % dropout rates for the third pentavalent dose, which is considered above acceptable limits in the expanded programme on immunization, were considered for inclusion in the study. In 2012, 34 districts with dropout rates more than 10 % were identified. Among these, districts with very high coverage rates (third dose pentavalent coverage ≥90 %) were excluded, as were districts that were geographically hard-to-reach or with security concerns. Six districts with dropout rates ranging from 13-27 % with both rural and urban settings were identified, and subjected to simple random sampling to select three districts. These districts included Machakos, Langata and Njoro (Fig. 1).

Machakos District is in Machakos County, covers an area of 6,281.4 kmsq, most of which is semi-arid with a projected population of 211,404 from 2009 census [26] and 13,271 live births in 2012. There are 56 health facilities, on average the distance to health facilities is 5 km, in 2012 the district achieved coverage of 88 % for the first dose of pentavalent, 79 % for the third dose, and 76 % full vaccination coverage. The dropout rate among children for the third dose of pentavalent vaccine was 13 % . Langata district is one of the districts in Nairobi County, the district is an area with glaring contrast in living standards, ranging from the plush homes of Karen and Langata to the sprawling Kibera slums, which are characterized by poor living standards with a projected population of 397,238 from 2009 census [26], live births of 12,402 and 97 Health facilities. In 2012 the district achieved coverage of 96 % for the first dose of pentavalent, 84 % for the third dose, and 82 % full vaccination coverage. The dropout rate among children for the third dose of pentavalent vaccine was 13 %. Njoro district is in Nakuru county, had an estimated population 100,000,7,904 [26], live births and 37 health facilities. The main economic activity agribusiness with a large proportion of the population having to travel for more than five kilometres to access the nearest health facility. In 2012, the district achieved coverage of 86 % for the first dose of pentavalent, 75 % for the third dose, and 74 % full vaccination coverage. The dropout rate among children for the third dose of pentavalent vaccine was 13 %. Vaccination outreach services are conducted in all the three district to reach hard to reach areas and engagement of mass media to pass information to promote timely vaccination of young children.

### Study population

Children <12 months of age who were brought to the selected vaccinating health facilities in the three districts for their first dose of pentavalent vaccine were recruited on a first come basis until the strategy-level target sample sizes was reached. Children whose mothers did not have a telephone number were excluded from the study. Dropout was defined as any child who failed to return for the third dose of pentavalent vaccine two weeks or more after the scheduled date.

### Evaluation

We selected three health facilities in each district, and randomly allocated each facility to one of the two interventions to provide short text messages or stickers reminding caretakers to return for second and third dose of pentavalent vaccines, or to serve as the control group, receiving no extra reminder messages and continue providing the next appointment date in the well-child booklet. Participants were conveniently enrolled in the selected health facilities until the strategy-level target sample sizes were reached.

Caretakers of participants in the SMS intervention group received two text reminders via SMS. Reminders were dispatched from an automated web based system two days before and on the day of the scheduled vaccination due date for the second and third dose of pentavalent vaccine. The first message reminded the parent of the next due date for the vaccination and which health facility to attend for vaccination. The second message reminded the caretakers that the actual due date was that day. The text messages were sent in Kiswahili and English. The sticker intervention group received two stickers at the time of enrollment which noted the day of the scheduled vaccination due date and the name of the health facility. Caretakers were instructed to place one sticker on the child’s health booklet, and the other sticker in a visible area of the main household or within the bedroom. Placement of the sticker within the home was verified during subsequent visits by asking the parent where they placed the sticker. The control group received no reminders, but the scheduled vaccination due date was indicated on the child’s health booklet as per routine procedures. All the groups received routine health education and advice on vaccination. Any caretaker who failed to return the child for vaccinations two weeks or more after the expected completion of third pentavalent dose was contacted by the investigator to establish reasons for missed vaccinations.

### Data collection and analysis

Data were collected by study nurse and principal investigator during routine working hours at the maternal child health clinic on a daily basis. Caretakers were interviewed face to face using a pretested standard questionnaire. The questionnaire collected information on socio-demographic, knowledge and source of information on vaccination, and recorded details of vaccines received during each visit.

Data were entered and analyzed using Epi info software. The primary outcome measure was receipt of scheduled vaccines at 10 and 14 weeks. The secondary outcome measures were dropout in vaccination and factors associated with missed vaccinations. We conducted data analysis using Epi Info version 7.1.4 and excel analysis software. Proportions and means were calculated for categorical and continuous variables respectively and summarized into tables and figures for univariate analysis. Bivariate and Multivariate analysis using unconditional logistic regression using facility clusters were conducted to identify independent predictors of missed vaccinations. Odds and Adjusted Odds Ratio (OR & AOR) and 95 % Confidence Interval (CI) were used to estimate the strength of association between independent variables and the dependent variable. The threshold for statistical significance was set at *p <* 0.05. We calculated dropout rate as a percentage of the difference between first and third pentavalent dose.

Sample size calculation was done using Casagrande et al. [27] formula for comparing two proportions to detect a 15 % decrease in the drop-out vaccination rate for each of the three intervention groups, assuming a dropout rate for the third dose of pentavalent of 15.6 % [28], study power of 80 %, and confidence level of 95 %. The minimum sample size was 372 participants per intervention arm.

## Ethical considerations

Written informed consent was obtained from caregivers of eligible children before enrolment. The study protocol was reviewed and approved by Kenyatta National Hospital/University of Nairobi Ethical Review Committee (P388/07/2013). Confidentiality of records was maintained constantly under lock and key system and will be destroyed five years after data collection is completed.

## Results

We assesses 1,126 children, Ten (0.9 %) were excluded due to lack of mobile phone number by parents. One thousand one hundred and sixteen children (1,116) were enrolled; 372 in each intervention group and 372 controls between February and October 2014. The median age of children was 45 days (range: 31–99 days), and 574 (51 %) were males. The mean age of caretakers was 26 years (14–45), 856 (77 %) were unemployed, 549 (49 %) had attained up to primary level education, 959 (86 %) owned mobile phones and 157 (14 %) used family members phone. There were no statistical differences in demographic characteristics among caretakers and children enrolled in each of the three groups (Table 1).

**Table 1:** Univariate analysis of socio-demographic characteristics of mothers/child attending vaccination services in Machakos, Njoro and Langata districts in Kenya, 2014

Variable	SMS n (%)	Sticker n (%)	No intervention n (%)	p-value
Sex				
Female	181(49)	170(46)	191(51)	0.3
Male	191(51)	202(54)	181(49)	
Child’s age				
≤ 42 days	53(14)	53(14)	51(14)	0.95
43–49 days	265(71)	260(70)	269(72)	
50-56days	32(9)	40(11)	32(9)	
57-63days	9(2)	10(3)	11(3)	
≥ 64 days	13(3)	9(2)	9(2)	
Maternal age				
≤ 20	66(18)	69(19)	47(13)	0.1
21–25 years	134(36)	151(41)	136(37)	
26–30	102(27)	85(23)	113(30)	
31–35	50(13)	42(11)	41(11)	
> 35 years	20(5)	25(7)	35(9)	
Maternal employment				
Employed	90(24)	80(22)	101(27)	0.2
Unemployed	282(76)	292(78)	271(73)	
Maternal education				
N o formal education	1(0)	2(1)	6(2)	0.09
Primary	151(41)	170(46)	175(47)	
Secondary	140(38)	118(32)	108(29)	
Tertiary	80(21)	82(11)	83(22)	
Marital status				
Married	303(81)	320(86)	322(87)	0.1
Single	69(19)	52(14)	50(13)	

**Fig. 2:**
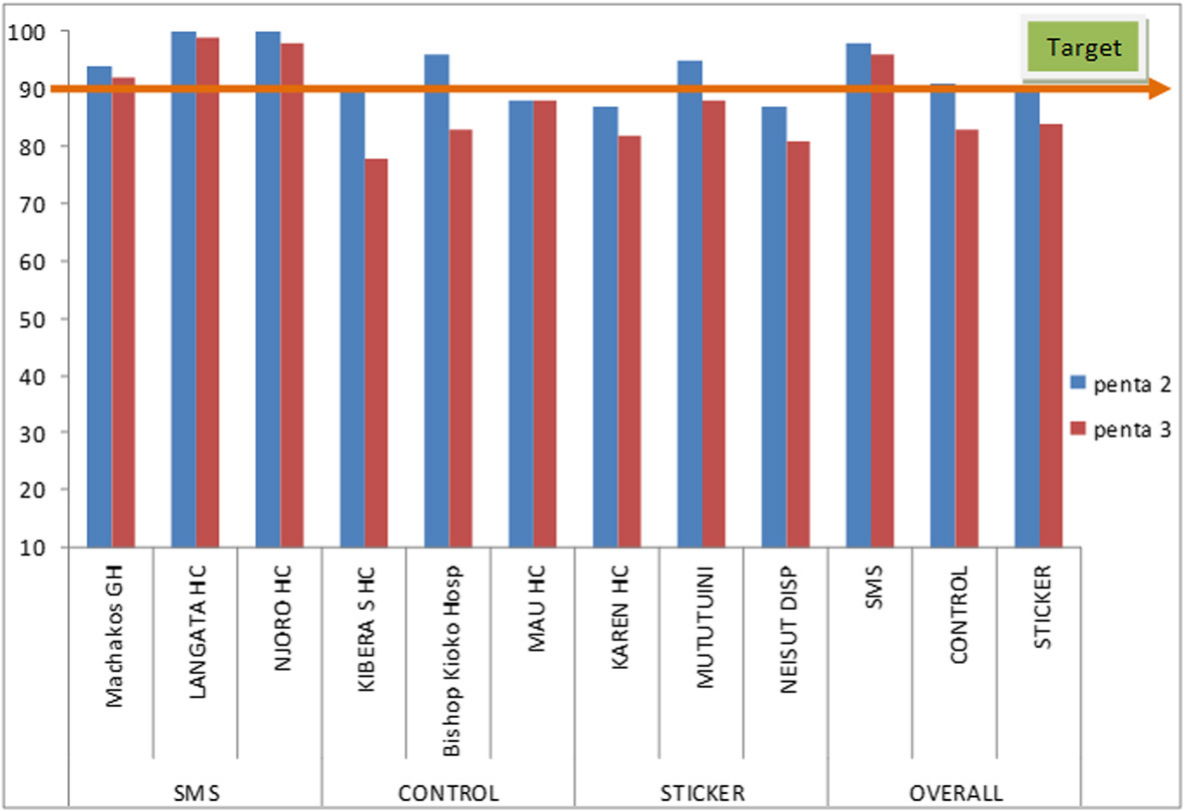
Vaccination coverage at 10 and 14 weeks by facility among children attending vaccination services in Machakos, Njoro and Langata districts, Kenya 2014

In the SMS intervention group, at 10 weeks of age 365 (98 %) children had received their second dose of pentavalent vaccine. At 14 weeks of age, 359 (96 %) had received their third dose of pentavalent vaccine (*p =* 0.4). In the sticker intervention group at 10 weeks of age, 334 (90 %) children had received their second dose of pentavalent vaccine. At 14 weeks of age 312 (84 %) had received their third dose of pentavalent vaccine (*p =* 0.02). In the control group at 10 weeks of age, 340 (91 %) children had received their second dose of pentavalent vaccine. At 14 weeks of age, 309 (83 %) of children had received their third dose of pentavalent vaccine (p ≤ 0.001) (Fig. 2). There was a significant increase in dropouts between second and third dose of pentavalent vaccine in the control and sticker intervention groups, but no significant increase in dropouts in the SMS intervention group. At 10 weeks, the risk difference for those who received SMS reminders and the control group was seven percent (95 % CI: 0.3–14). At 14 weeks, the risk difference for those who received SMS reminders and the control group was 13 % (95 % CI: 5.6–21.26). The mean delay in receiving second dose of pentavalent vaccine on the scheduled date in the SMS intervention group was 0 days (standard deviation (SD): 1.2), in the control group the mean delay was one day (SD: 4.3), while in the sticker group, the mean delay was one day (SD: 6.3). There was a significant difference in the mean delay in days between the SMS and Control group (*p <* 0.001), but no significant difference in delay between the control and sticker group (*p =* 0.5). The mean delay in receiving the third pentavalent dose on the scheduled date in the SMS intervention group was 0 days (SD: 2), in the control group, two days (SD: 7) and in the sticker group, two days (SD 6). There was a significant difference in the mean delay in days between the SMS and Control group (*p <* 0.001), but no significant difference in mean delays in days between the control and sticker group (*p =* 1).

A total of 1,488 messages were sent to the participants in the SMS group, cost $33.1 USD, and the premium cost of scheduling messages from web for six months cost $66.7 USD giving a cost of $0.27 USD per child for the project. Overall, among those children enrolled who received the first dose of pentavalent vaccine, 136 (12 %) did not return for their third dose of pentavalent vaccine. Of these, 63 (17 %) were from the control group compared to 13 (4 %) from the SMS intervention group (OR 0.2, CI:0.04–0.8), and 60 (16 %) were from the sticker intervention group (OR: 0.94, CI: 0.53–1.6).We traced 110 (81 %) caretakers to identify reasons for missed vaccination that included: child taken to another facility 39 (35 %); travelled out of town 33 (30 %); forgot 17 (15 %); child was sick 16 (15 %); or child died 2 (2 %).

On bivariate analysis, those who received SMS reminders (OR 0.2, CI 0.04–0.8) were 20 % less likely to miss vaccinations. In contrast, an education level of primary and below (OR 1.9, CI 1.1–3.3), age of child at first pentavalent dose >56 days (OR 2.2, CI 1.3–3.1), residing a distance ≥5 km from facility (OR 1.6, CI :1.1–2.3), waiting time >30 min (OR 1.4, CI 1.0–2.1) were associated with higher odds of missed vaccinations (Table 2).

**Table 2:** Bivariate analysis of factors associated with missed vaccination among children attending vaccination services in Machakos, Njoro and Langata districts in Kenya, 2014

Variable	Dropout	No dropout	OR(CI)
	n(%)	n(%)	
Mother Age			
<25 years	74 (12)	528 (88)	1.0 (0.7–1.4)
>25 years	62 (12)	451 (88)	
Education			
Primary and below	82 (15)	476 (85)	1.9 (1.1–3.29)
Secondary and above	17 (8)	188 (92)	
Marital status			
Not Married	21 (13)	138 (87)	1.1 (0.67–1.82)
Married	113 (12)	822 (88)	
Place of Delivery			
Home	25 (14)	153 (86)	1.2 (0.75–1.89)
Hospital	111 (12)	824 (88)	
Employment			
Unemployed	29 (11)	231 (89)	0.9 (0.57–1.36)
Employed	107 (13)	749 (87)	
Age of child at penta 1			
>56 days	20 (22)	70 (78)	2.2 (1.3–3.8)
<56 days	116 (11)	910 (89)	
Distance from facility			
>5 km	64 (15)	354 (85)	1.6 (1.095–2.26)
<5 km	72 (10)	626 (90)	
Birth order			
First born	52 (12)	377 (88)	0.9 (0.69–1.43)
Not a first born	84 (12)	603 (88)	
Waiting time			
>30Mins	65 (15)	378 (85)	1.4 (1.03–2.12)
<30Min	70 (10)	601 (90)	
Transport paid			
Yes	63 (12)	464 (88)	0.96 (0.67–1.38)
No	73 (12)	516 (88)	
Interventions			
SMS reminder	13 (3.5)	359 (96.5)	0.2 (0.04–0.8)
No reminder(control)	63 (17)	309 (83)	
Control	63 (17)	309 (83)	0.94 (0.53–1.67)
Sticker reminder	60(16)	312(84)	

In multivariate analysis, mothers with maternal education level of primary and below (OR 1.9, CI: 1.0–2.7), and residing >5 km from a health facility (OR 1.6, CI 1.1–3.1) were more likely to dropout. In contrast, those who received SMS reminders were 10–40 % less likely to miss vaccinations in comparison to the control group (OR 0.2, CI: 0–0.8). (Table 3).

**Table 3:** Multivariate analysis of factors associated with missed vaccination among children attending vaccination services in Machakos, Njoro and Langata districts in Kenya, 2014

Variable	AOR	95 % CI	P-Value
Child’s age >56 days	1.81	0.83–3.90	0.14
Education level below primary	1.85	0.99–2.70	0.05
Distance >5 km from facility	1.64	1.09–3.10	0.025
Waiting time > 30 min	0.86	0.51–1.45	0.57
SMS reminder	0.196	0.09–0.43	<0.001
Sticker Reminder	0.69	0.40–1.20	0.181

## Discussion

This evaluation with a large number of participants found that SMS reminders were effective in reducing dropouts for vaccinations in the selected districts in Kenya. The vaccination coverage was significantly higher amon those receiving SMS reminder than those receiving routine reminders. About 13 % of the children vaccinated in the SMS intervention group is attributed to SMS reminders who likely would not have been vaccinated if SMS reminders had not been used at 14 weeks. This finding is similar to a study conducted in Kadoma city in Zimbabwe (2013) that demonstrated high vaccination coverage among those who received SMS reminders [24]. A systematic review of effects of all types of reminders including SMS found that patient reminder systems were effective in improving vaccination rates [29]. Also studies conducted in low-income, minority populations in New York City found that SMS reminders improved coverage from 4 % to 17 %, depending on the vaccine [13]. Although the mean delay of one day in receiving the second dose of pentavalent vaccine and two days in receiving the third dose of pentavalent vaccine for the control group compared to the SMS text reminder group which had no delays for the provision of these doses of pentavalent vaccine was highly statistically significant, in our opinion a delay of one or two days is clinically not significant in terms of susceptibility to disease. Thus our findings support the hypothesis that SMS is an effective reminder system for vaccination services.

We also found that there was no difference between the sticker reminders group and the control group. The vaccination coverage at 10 and 14 weeks were not statistically significant between the sticker reminder and the control group. A study done in Ethiopia in 1993 found stickers to be effective in reducing vaccination dropouts [6]. However,, unlike our study, the control group in the Ethiopia study used a population that had been vaccinated during the previous year. Additionally, children whose mothers had below secondary level education and children residing >5 km from the health facility was associated with being a drop-out. Similar finding were detected in previously conducted studies in Kenya [28, 30–33]. These data suggest that while efforts are needed to find effective methods for vaccination reminders, the access factors (distance from health facility) remain a challenge.

This study is subject to several limitations. If a care giver took the child to another facility for second or third pentavalent dose, the system considered the child unvaccinated,leading to misclassification, however, a sensitivity analysis that assumed that these children were actually vaccinated had no effect on the general observed difference between the inteventions. The results of this study may not be generalizable for the entire population in the country.

## Conclusions

Vaccination coverage was higher in the SMS intervention group than in the control group; this result was both statistically and clinically significant. The overall increase may be attributed to the use of SMS reminders in this study. The difference in coverage between the sticker intervention group and the control group was not statistically significant, and may be an indication of the ineffectiveness of sticker reminders.

We recommend the extended implementation of SMS reminders in routine vaccination services in Kenya.
